# Response of Knee Extensor Muscle-Tendon Unit Stiffness to Unaccustomed and Repeated High-Volume Eccentric Exercise

**DOI:** 10.3390/ijerph18094510

**Published:** 2021-04-23

**Authors:** Pornpimol Muanjai, Mantas Mickevicius, Audrius Snieckus, David A. Jones, Pavelas Zachovajevas, Danguole Satkunskiene, Tomas Venckunas, Sigitas Kamandulis

**Affiliations:** 1 Department of Physical Therapy, Allied Health Sciences Faculty, Burapha University, Chonburi 20131, Thailand; 2 Exercise and Nutrition Innovation and Sciences Research Unit, Burapha University, Chonburi 20131, Thailand; 3 Institute of Sport Science and Innovations, Lithuanian Sports University, 44221 Kaunas, Lithuania; Mantas.Mickevicius@lsu.lt (M.M.); Audrius.Snieckus@lsu.lt (A.S.); jonesda14@gmail.com (D.A.J.); danguole.satkunskiene@lsu.lt (D.S.); Tomas.Venckunas@lsu.lt (T.V.); Sigitas.kamandulis@lsu.lt (S.K.); 4 School of Healthcare Sciences, Manchester Metropolitan University, Manchester M15 6BH, UK; 5 Department of Applied Biology and Rehabilitation, Lithuanian Sports University, 44221 Kaunas, Lithuania; Pavelas.Zachovajevas@lsu.lt

**Keywords:** muscle pain, stiffness, eccentric exercise, repeated-bout effect, knee extensor muscle

## Abstract

The purposes of this study were to investigate the muscle-tendon unit stiffness response and to compare the stiffness with those of other indirect markers induced by two bouts of unaccustomed eccentric exercise. Eleven untrained men performed two bouts of 200 maximal eccentric contractions of the right quadriceps 4 weeks apart. Changes in stiffness, pain evoked by stretching and pressure, plasma creatine kinase (CK) activity, and muscle thickness were followed for 7 days after each bout. Stiffness and pain peaked immediately and 1 day after the first exercise bout, whereas CK and thickness were highest 4 and 7 days after the first exercise bout, respectively (*p* < 0.05 for all). Muscular pain, thickness, and stiffness responses were lower by 53.3%, 99%, and 11.6%, respectively, after the repeated bout compared to after the first bout (*p* < 0.05 for all), while CK activity response did not differ significantly between bouts. High responders for an increase in muscle-tendon unit stiffness showed a repeated-bout effect for stiffness, pain, and CK activity (by 29%, 65%, and 98%, *p* < 0.05 for all), but the repeated-bout effect was not that clear in low responders. These findings suggest that a repeated eccentric exercise bout effect on stiffness in quadriceps is mostly not associated with muscle pain and CK activity, but there are large individual differences.

## 1. Introduction

Muscle stiffening and delayed-onset muscle soreness (DOMS) are typical sensations experienced soon after unaccustomed eccentric exercise [1]. Although the mechanisms and origin of the specific triggers of muscular stiffness are not clearly understood, tissue swelling is thought to be one direct cause [2]. The asynchronous development of swelling and stiffness suggests that they may not be causally linked [3]. Our recent study showing that postexercise stiffness develops much earlier than swelling [4] provided evidence that questions the early claim that these two processes are interrelated and instead suggested that they are likely to be two aspects of the same phenomenon. There are other possible causes, such as increased intracellular calcium concentration and contracture of damaged portions of muscle fibers [2,3]. In our earlier study of the interrelationships between muscle-tendon unit (MTU) stiffness, muscle pain and tenderness, swelling, and plasma CK release after acute eccentric exercise of the elbow flexors, we found strong correlations between muscle pain in response to stretching and increased resting elbow angle, and between peak plasma CK activity and the extent of swelling [4].

It is of practical importance that both muscle stiffness [4,5] and pain [4,5,6] responses are reduced when the exercise is repeated after the recovery from the previous bout. This phenomenon is called the repeated-bout effect (RBE) and is documented in both humans and animal models [7]. Several studies have demonstrated the protective effects of a previous exercise session on arm stiffness [5], leg stiffness [8], arm pain [5], leg pain [6], and muscle CK release [9] following the repeated bout. However, study results relating to whether there is an RBE on EIMD markers and MTU stiffness are inconsistent. For instance, stiffness of the plantar flexor MTU was not lower after a repeated exercise bout compared with the first bout [6]. By contrast, Janecki et al. [10] found less elbow flexor MTU stiffness following a repeated bout of 30 eccentric contractions. These differences may reflect differences in the EIMD induced by the exercise protocols, which used different combinations of exercise parameters such as volume and intensity. It may also be relevant that muscles of the upper limbs are much more susceptible to EIMD than are lower-limb muscles [11].

In the current study, we aimed to examine the dynamics of active stretch-induced muscle pain, pressure-evoked pain, swelling, CK release, and passive MTU stiffness after the first and repeated bout of eccentric exercise of the knee extensors. As a proxy of stiffness, the resistance of the passively stretched MTU was measured on an isokinetic dynamometer as has been used by others [12]. To compare indirectly the different aspects and time course of the responses to acute and repeated damage-evoking exercise between different muscle groups (lower vs. upper body), we used a protocol for high-volume eccentric lower-limb exercise to induce muscle damage of a magnitude comparable with that used to induce damage to the arm muscles [4]. We hypothesized that the reduced response of leg muscle stiffness after the second session because of the RBE would be related to the reduction in pain, which would confirm associations recently reported for arm exercise [4].

## 2. Materials and Methods

### 2.1. Participants

Eleven recreationally active men (mean ± standard deviation (SD); age, 21.3 ± 2.0 years; body weight, 81.5 ± 14.3 kg; height, 180.2 ± 3.5 cm) volunteered for the study. In order to assure that young, healthy, and non-familiar with eccentric types of activities, participants were recruited before being enrolled in the study who were asked about their age, health status, and physical activity prior to the study. Participants were included if they were 18 to 25 years, healthy, and not engaged in any type of resistance exercise during the previous 6 months. The exclusion criteria were the inability to abstain from strenuous physical activity during the entire study duration, any neuromuscular or skeletal problem, and the use of analgesic or anti-inflammatory drugs. The required minimum sample size was calculated before the study based on the expected passive knee extensor stiffness difference of 10% between exercise bouts, with an alpha level of 0.05 and the desired power of 80%. The calculation provided a sample size of at least ten participants.

Before participating in the study, the volunteers provided their written informed consent. The study conformed to the standards set by the latest revision of the Declaration of Helsinki and was approved by the Kaunas Regional Biomedical Research Ethics Committee (registration no. BE-2-17).

### 2.2. Study Design

The participants were familiarized with the equipment and measurements on the separate visit to the laboratory 3–4 days before the first exercise bout intervention. During the testing session, the following variables were measured before, immediately after, and 1, 2, 4, and 7 days after the exercise intervention: stretch- and pressure-induced muscle pain, MTU passive stiffness, swelling of vastus lateralis (VL) muscle as a change of muscle thickness, and plasma CK activity. The exercise intervention involved 200 maximal-effort eccentric isokinetic contractions of the knee extensors of the right leg performed twice, with a 4-week break between bouts. All exercise and testing was carried out by the same team of investigators. The temperature in the laboratory was kept stable between 20 and 22 °C.

### 2.3. The Intervention Exercise for Knee Extensors

The participant was seated on an isokinetic dynamometer (Biodex System 3 Biodex Medical Systems, Inc., Shirley, NY, USA) with the backrest fixed at 90° and with the trunk, pelvis, and right thigh stabilized to the chair of the dynamometer with Velcro straps. The axis of rotation was fixed at the knee joint, and the lever arm pad was attached 2–3 cm above the ankle. The participant was instructed to perform 20 sets (with 1 min breaks for recovery between sets) of 10 maximal voluntary eccentric contractions of the knee extensors in the range of 30–110° of knee flexion (0° = full knee extension) with the angular velocity set at 90°∙s^−1^. The participant was instructed to exert a maximal-effort contraction of the knee extensors (resisted knee flexion) through the full range of motion (ROM). Between each contraction, the leg was fully relaxed for about 1 s to allow the machine to return the leg to the starting position at 30° knee flexion. Verbal encouragement to maintain maximal effort was given for each contraction. Torque was monitored throughout the 20 sets of exercises for the two exercise bouts.

### 2.4. Muscle Pain and Tenderness

Pain elicited by taking the knee to full flexion at 125° (stretched pain) during the endpoint of the passive stiffness test was evaluated using a visual analog scale of 0–10, where 0 represented “no pain” and 10 “intolerably intense pain” [13]. The pressure pain threshold (PPT) used to evaluate tenderness of knee extensors was measured with an algometer (1 cm diameter probe, Wagner Instruments, Greenwich, CT, USA) with the participant lying on the examination table with the knee extended. The probe was applied above the patella at a position 50% to a line between the anterior superior iliac spine and the superior border of the patella, laterally and centrally, for the VL and rectus femoris (RF), respectively. The probe was applied 10% to the reference line medially for the vastus medialis (VM). During the measurement, the investigator applied the tip of the device perpendicular to the skin and slowly increased pressure to 30 N. The participant reported the endpoint when the pressure first became a painful sensation. The applied force at the endpoint of the two trials was averaged and subtracted from 30 [4] and is referred to here as “PPT pain”. The three measurement sites were firstly marked with a permanent marker with the guidance of real-time B-mode ultrasound, with the largest part of each muscle visible for repeated testing.

### 2.5. Passive Resistive Torque and MTU Stiffness of the Knee Extensors

To measure passive resistive torque, the participant was positioned lying on his back in a padded chair of the isokinetic dynamometer without shoes and with the right leg straight and parallel to the floor. The left leg was fully flexed at the hip and knee joints and held by the therapist to limit lumbopelvic motion (adapted from the work of Krause et al. [14]). The pelvis and the right thigh were fastened to the chair with Velcro straps. The axis of rotation of the right knee was fixed to the fulcrum of the dynamometer shaft with the knee out of the chair approximately by the distance of the 3 finger bases to ensure sufficient space for knee flexion to 125° without any lever arm strain, and the lever arm pad was fastened proximally to the malleolus. All dynamometer chair settings were noted for reproducibility. In this configuration and with the participant fully relaxed, the dynamometer passively flexed the knee to 125° at 5°∙s^−1^. Passive stiffness was subsequently determined from the slope of the curve derived for the passive torque to angle relationship (with gravity correction for the weight of the leg). A third-degree polynomial using the least-squares method (R^2^ value was used to evaluate the fit) was fitted to the linear portion of the curve for 85–100% of the maximal knee flexion. Measurements were accepted only if the knee muscle activity of the extensors (RF and VL) and flexors (biceps femoris muscle) was both <5% of the maximal electromyographic values [15], which had been predetermined by having the participant perform a maximum voluntary isometric contraction to obtain the peak of the root mean square. Three passive maximum peak torque and stiffness values were averaged for further analysis. The intraclass correlation coefficient for stiffness was 0.974 (95% confidence interval (CI): 0.904–0.993) in this study.

### 2.6. VL Muscle Thickness

Between the examinations of muscle pain and PPT pain, transverse images of the VL mid-belly were obtained using B-mode ultrasonography with a 10–15 MHz transducer (Echoblaster 128, UAB; Telemed, Vilnius, Lithuania) with minimal probe pressure application. The clearest images of the fascia captured were analyzed, and the probe placement was outlined with a permanent marker at the mid-belly in the same point for PPT of VL measurement. VL muscle thickness was then measured between the superficial and deep aponeurosis [16] using ImageJ image analysis software (Wayne Rasband, NIH, Bethesda, MD, USA). In this study, the intraclass correlation coefficient for muscle thickness was 0.895 (95% CI: 0.845–0.923). The average thickness from two VL images was used in further analyses.

### 2.7. Plasma CK Activity

About 0.25 mL of capillary blood was drawn from the finger and immediately centrifuged, and the plasma was used for measurement of CK activity using a bench-top biochemical analyzer [4] (Spotchem^TM^ EZ SP-4430, Menarini Diagnostics, Winnersh-Wokingham, UK) using soft reagent strips (Arkray Factory, Inc., Shiga, Japan).

### 2.8. Statistical Analysis

Descriptive data are presented as mean and SD; the standard error of the mean (SEM) is given where means are compared. The Shapiro–Wilk test was used to check whether the data were normally distributed. When not distributed normally, the data are reported as the median and interquartile range (IQR). Peak torque and work performed during the eccentric exercise were compared using paired *t*-tests. A repeated-measures analysis of variance was used to compare the change in MTU stiffness using the following parameters: condition (first or repeated bout) × time (before, immediately after, and 1, 2, 4, and 7 days after exercise). When significant main effects were found, post hoc testing was performed using paired *t*-tests with the Bonferroni correction for multiple comparisons. Partial eta-squared was used as a measure of the effect sizes (ES) in repeated measurements statistics. The data that failed the test for normal distribution were analyzed using the nonparametric Wilcoxon’s signed-rank and Mann–Whitney *U* tests.

The relationships between the two measures of the change in stiffness, pain, plasma CK activity, and muscle thickness were identified using linear regression. The correlation coefficient (r) was used to identify the explained variance between tests. Significance was accepted as *p* < 0.05. As in our previous study on upper body exercise [17], the response to eccentric knee extension exercise was analyzed both on a whole group level as well as to more comprehensively represent the variability of the individual response, mainly for stiffness in this study, separately in low (*n* = 4) and high responders (*n* = 4) for MTU stiffness response to the first exercise bout. The test-retest reliability of the selected variables was found using the absolute agreement intraclass correlation coefficient (ICC 2,1) with a 95% confidence interval (CI) between the familiarization session and the baseline before first bout scores. Statistical analysis was performed using IBM SPSS Statistics for Windows (version 23.0, IBM Corp., Armonk, NY, USA).

## 3. Results

### 3.1. Eccentric Exercise

All participants successfully completed the two exercise sessions of 200 eccentric contractions. Peak torque declined progressively across the sets of 10 contractions to 55.1% ± 14.2% of the value in the first set of the first bout and to 69.0% ± 13.4% during the repeated bout (both *p* < 0.001) and did not differ significantly between bouts (*p* > 0.05). Similar dynamics were evident in the change in work from the first to the last set, which decreased to 62.4% ± 14.5% during the first bout and to 71.1% ± 14.2% during the repeated bout (both *p* < 0.001) with no difference between bouts (*p* > 0.05).

### 3.2. Repeated-Bout Effect

**Figure 1:**
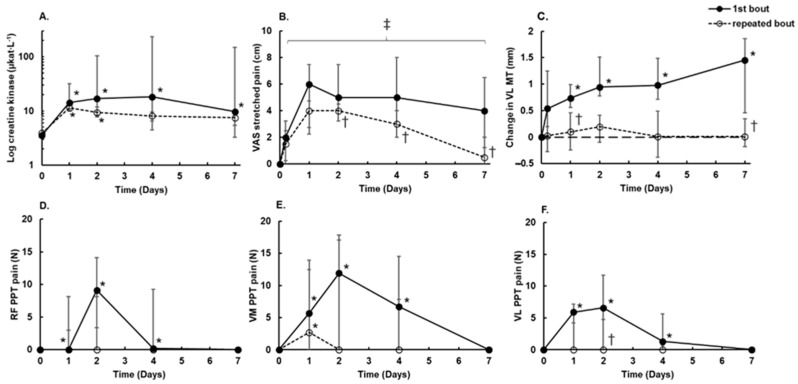
Responses to a repeated bout of 200 knee extension eccentric contractions. (**A**) Plasma creatine kinase (CK) activity expressed on a log scale. (**B**) Visual analog scale (VAS) to rate stretched muscle pain. (**C**) Thickness of vastus lateralis (VL). (**D**) Pressure pain threshold (PPT) in rectus femoris (RF). (**E**) PPT pain in vastus medialis (VM). (**F**) PPT pain in VL. Data are presented as the median and interquartile range (IQR) (*n* = 11). Filled circles and solid lines are data for the first bout; open circles and dashed lines are data for the repeated bout. * Significant difference from before exercise (*p* < 0.05). ^‡^ Significant difference from before exercise for both exercise bouts (*p* < 0.05). ^†^ Significant difference from the first exercise bout (*p* < 0.05).

The differences in the responses of markers of EIMD after the first and repeated bouts are shown in Figure 1. Plasma CK activity peaked around day 4 being median 18.1 µkat (IQR: 13.7–216.5 µkat, *p* = 0.017) and was elevated for 7 days after the first bout, whereas it was elevated for only 1–2 days (11.3 µkat, IQR: 9.97–13.16 µkat, *p* < 0.05) after the repeated bout (Figure 1A). In 7 of 11 participants, CK activity was 5–200 times lower after the repeated bout. In the other four participants, a low CK activity response was evident after the first bout, which left little room for a decrease by several folds after the repeated bout. The difference between bouts did not reach significance because of this high variability in the CK response.

The stretched pain in the damaged muscle was maximal 1 day after the exercise. The median pain ratings were 6 points (IQR: 2.31–7.53 points) after the first bout and 4 points (IQR: 2.80–4.81 points) after the repeated bout (*p* = 0.029). Stretched pain in the damaged muscle remained evident during the 7 days after both exercise bouts. The RBE on pain development was seen on days 2–7 after the repeated exercise (*p* < 0.01 for all) (Figure 1B).

VL muscle thickness increased progressively in response to exercise and reached a peak by day 7 after the first exercise bout (F(1,9) = 12.1, *p* = 0.007, ES = 0.57). However, no increase was evident after the repeated exercise bout (*p* > 0.05) (Figure 1C).

Exercise-induced muscle tenderness, as measured by increased PPT pain in the RF, VM, and VL muscles (Figure 1D–F), increased over days 1 to 4, peaked on day 2 (*p* < 0.05 for all muscles) after the first bout, and had recovered fully by day 7. The repeated bout induced tenderness only in the VM muscle 1 day after exercise (*p* = 0.028) (Figure 1E). The maximum pressure pain observed after the first bout of exercise did not differ between the three muscles (*p* > 0.05).

**Figure 2:**
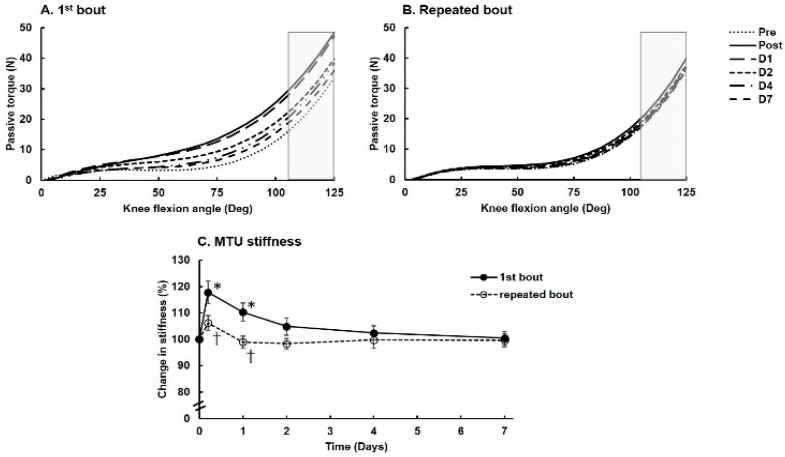
Changes in passive MTU stiffness of the knee extensors. Angle–torque relationships for (**A**) the first bout and (**B**) the repeated bout. The gray shaded boxes indicate the stiffness calculation for the last 15° (110–125°) of passive knee flexion. (**C**) Passive MTU stiffness after 7 days is shown as mean and SEM (*n* = 11). Filled circles and solid lines are data for the first bout; open circles and dashed lines are data for the repeated bout. * Significantly different from the pre-exercise value (*p* < 0.05). ^†^ Significantly different from the first exercise bout (*p* < 0.05).

Data for MTU stiffness of the knee extensors are shown in Figure 2. The slope of the passive torque at the last 15° of knee flexion responded differently between the two exercise bouts (Figure 2A,B). MTU stiffness reached a peak at 17.8% (CI: 6.9–28.6%) above the baseline value immediately after the first bout (F(5,40) = 5.8, *p* < 0.001, ES = 0.42) and at 6.1% (CI: 1.3–13.6%) above the baseline immediately after the repeated exercise bout (*p* > 0.05, Figure 2C). MTU stiffness remained higher on day 1 after the first exercise bout but not on day 1 after the repeated bout (bout effect for pre-, post- and 1 day postexercise values, F(1,8) = 6.3, *p* = 0.036, ES = 0.44). On days 2–7, MTU stiffness did not differ from the baseline value or between exercise bouts (Figure 2C). Peak resistive torque at the endpoint of passive knee flexion was lower after the repeated compared to first bout (F(1,8) = 6.2, *p* = 0.038, ES = 0.44) (Figure 2B).

### 3.3. Correlations between MTU Stiffness and Markers of EIMD

Baseline MTU stiffness did not correlate with changes in any of the markers of EIMD (*p* > 0.05) (Table 1). There was a significant, but modest correlation between peak stiffness and peak muscle swelling (r = 0.50, *p* = 0.04) but not between peak stiffness and peak pain (r = 0.18, *p* > 0.05) or peak plasma CK activity (r = 0.33, *p* > 0.05) (Table 1).

### 3.4. High Responders vs. Low Responders

**Figure 3:**
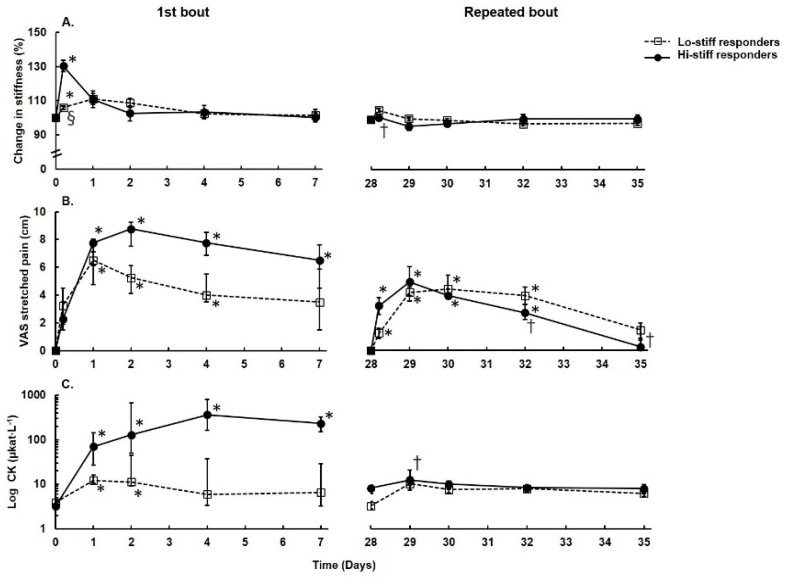
Variable responses to the repeated bout identified as the change in passive MTU stiffness. Filled circles and solid lines are the data for the highest stiffness response (“high responders,” *n* = 4); open circles and dashed lines are the data for the lowest stiffness response (“low responders,” *n* = 4). (**A**) Change in passive MTU stiffness. (**B**) Visual analog scale score for the pain of stretched muscle. (**C**) Plasma creatine kinase (CK) activity on a log scale. Data are presented as the median and interquartile range (IQR) except for stiffness data, which are presented as the mean and standard error of the mean. * Significant difference from the pre-exercise value (*p* < 0.05). ^†^ Significant difference from the first exercise bout (*p* < 0.05). ^§^ Significant difference between high and low responders (*p* < 0.05).

The stiffness, VAS score, and plasma CK activity of the four participants with the highest MTU stiffness (30.3%, CI: 13.1–47.6%, *p* = 0.011) and four participants with the lowest MTU stiffness (5.9%, CI: 0.5–11.3%, *p* = 0.04) immediately after the first exercise bout are presented in Figure 3. MTU stiffness did not change after the repeated bout in any group (*p* > 0.05) (Figure 3A). Stretched pain in the exercised muscle increased over days 1–7 after the first bout (*p* < 0.05) but was much lower (*p* < 0.05) on days 4–7 after the repeated bout for the high responders (Figure 3B). The pain did not differ between the bouts in low responders (*p* > 0.05). Plasma CK activity in the high responders tended to be higher and last longer over the 7 days compared with that in the low responders, for whom it increased nonsignificantly only 2 days after the first exercise bout (*p* > 0.05) (Figure 3C). Plasma CK activity after the repeated bout was reduced on day 1 after exercise (*p* = 0.043, compared to the first bout) in the high responders, but no training effect was noted for the low responders (*p* > 0.05). VL muscle thickness did not differ between high and low responders independently of the exercise bout (*p* > 0.05 for all comparisons).

## 4. Discussion

The primary purpose of this study was to examine the dynamics of exercise-induced muscle pain, swelling, CK release, and passive stiffness after the first and repeated bout of eccentric exercise of the knee extensors. The secondary aim was to indirectly compare the different aspects of the response to acute and repeated damage-evoking exercise between lower vs. upper bodymuscle groups as the current study applied a protocol for high-volume eccentric leg exercise to induce muscle damage of a magnitude comparable with that for the arm muscles in the previous study [4]. The results of the present study, in which 200 maximal-effort eccentric contractions of the knee extensors were used, have confirmed the major RBE on the stretched pain, pressure pain threshold (tenderness), and swelling in the exercised muscle. However, the protective effect against exercise-induced MTU stiffness was small and was not evident for the change in plasma CK activity, mainly because both of these markers had large interindividual variability. The high responders for MTU stiffness, however, exhibited a more pronounced response for muscle pain and plasma CK activity after the first exercise bout. In addition, the induction of changes in all three markers was blunted after the repeated exercise bout for the high responders. Even though to a small degree, the results of the current study additionally support the link between stiffness, pain, and CK activity as observed previously in a study on elbow flexors [4].

The peak knee extensor MTU stiffness was detected already immediately after the first eccentric exercise bout and lasted for 1 day, after which it gradually resolved within 7 days. This pattern corresponds with that of peak gastrocnemius stiffness 24 h after exercise [18]. The change in peak passive torque measured immediately after exercise may be related to an acute increase in the water content within the muscle after highly metabolically demanding exercise [19]. We have noted a large variation of the changes in MTU stiffness in response to exercise. Variability in the extent of EIMD, as reflected by differences in muscle force loss, increased plasma CK activity, muscle soreness, swelling, and reduced ROM (increased MTU stiffness) may well have a genetic component, which may be reflected in the different individual responses in exercise-induced upregulation of heat shock proteins [20]. Regarding methodology for MTU stiffness, in our study, it depended on the properties of many structures, including agonists, antagonists, and noncontractile connective tissue, rather than providing a direct measure of the stiffness of the specific muscle [21].

There are several possible explanations of the origin of stiffness associated with EIMD. These encompass the release of Ca^2+^ because of membrane damage during eccentric exercise, increase in Ca^2+^ entry [3] and activity of calpains via stretch-activated channels [22], and changes in titin secondary to effects on the calcium-dependent pathway [23]. In the current study, we did not examine the precise mechanisms underlying the development of MTU stiffness after exercise. However, we found a relationship between peak stiffness and peak muscle swelling, although this does not seem to be causal because the markers had very different dynamics, as has been observed in elbow flexors [4].

In the present study, MTU stiffness in leg muscles lasted a shorter time compared with the exercise-induced stiffness observed in elbow flexors, which did not return to baseline until ~2 days after the bout of eccentric exercise [4]. The different responses of the upper vs. lower limbs might be explained by the extent of muscle damage evoked because the arm muscles are more susceptible to EIMD [11]. The plasma CK activity response was four times higher in a study of elbow flexors [4] than observed in the knee extensors in the present study. This difference implies that greater damage can be induced with only 60 maximal eccentric contractions of the smaller arm flexors compared with the 200 eccentric contractions performed by the large musculature of the leg extensors in our study. Other studies have also reported greater susceptibility to damage in the arm than leg muscles in young adults performing exercise at the same relative intensity [24].

With elbow flexors eccentric exercise, we have previously reported significant RBE for ROM, upper arm circumference, and stretch-induced pain, while only a small RBE for plasma CK activity and pressure-induced pain had been detected [4]. A magnitude of the protection of the repeated exercise bout, a “protective index”, could be calculated for each marker of muscle damage as a difference between the response to the first and repeated bout divided by the value of the response to the first bout and multiplied by 100; as such, a higher number of the protective index means a larger protective effect. The protective effect of the first bout for pain was ~50% for the arm muscles in our previous study [4], and it was ~33% for the leg muscles in the current study. These findings are consistent with those of Chen et al. [25], who found no significant difference between various muscle groups in the magnitude of the protection against soreness after the repeated exercise bout of 50 eccentric contractions. Notwithstanding, the protective effect of the first exercise bout against muscle soreness was reported as larger for elbow flexors than for knee extensors [11], and the protective effect of the first bout was 99–100% for plasma CK activity, which contrasts with the findings of our studies. These differences may reflect the high response variability between individuals.

The increase in MTU stiffness in this study was less substantial after the repeated than the first bout, as a similar finding to Margaritelis et al. [8]. Pincheira et al. [6] did not observe an RBE for exercise-induced stiffness and postulated that RBE is likely to be associated with noncontractile elements of the muscle rather than changes in mechanical factors or neural activation. The MTU stiffness response to eccentric exercise differed substantially between individuals in our study (Figure 3). It is notable that the high responders for MTU stiffness exhibited an RBE for MTU stiffness, stretch-induced pain, and plasma CK activity. By contrast, the low responders did not exhibit an RBE for any of the measured variables. These results here are largely consistent with previous findings that slow muscle recovery following eccentric exercise is characteristic in high responders and reflects mainly secondary damage, as indicated by swelling and increased CK activity, and that the faster recovery following a repeated bout occurs because of suppressed secondary damage response [17].

The major limitation of the current study is small sample sizes, whereas this corresponds with those in other similar studies where the numbers of participants ranged between 7 and 18 [1,26]. There was, however, a lack of statistical power in subgroup analyses that were used to present the typically high variability of response levels as presented in Figure 4 of our previous study [17]. The other limitation of the present study was that the knee extensors were investigated as the whole complex of contractile and adjacent connective tissues rather than by measuring the passive tension of only the muscle fibers. The advantages of using shear wave elastography might be considered when measuring the response of stiffness of a muscle or tendon unit to eccentric exercise. Using shear wave elastography, muscle stiffness of the elbow flexors when tested at long length (160°) peaked at 1 h after exercise that induced EIMD and persisted up to 21 days after exercise [27].

## 5. Conclusions

In summary, we have reported the RBE for MTU stiffness of the knee extensors in response to high-volume intense eccentric exercise. Changes in MTU stiffness were minor associated with muscle pain and plasma CK activity. However, there were large individual differences, which may also explain why RBE for MTU stiffness is difficult to detect at a group level.

## Figures and Tables

**Table 1:** Correlations between stiffness and peak pain in response to stretching with peak pain to pressure, swelling, and plasma CK activity (*n* = 11).

	Initial Stiffness		Peak Stiffness		Peak Pain on Stretch	
	r	*p*	r	*p*	r	*p*
**Peak pain on stretch**	−0.18 (−0.06,0.03)	0.47	0.18 (−1.91,3.83)	0.49	-	-
**Peak RF PPT**	−0.29 (−0.02,0.01)	0.25	0.42 (0.34,1.68)	0.09	0.30 (−0.06,0.24)	0.22
**Peak VM PPT**	−0.20 (−0.01,0.01)	0.43	0.35 (−0.22,1.23)	0.16	0.59 (0.05,0.27)	0.01
**Peak VL PPT**	−0.06 (−0.02,0.02)	0.82	0.35 (−0.38,2.11)	0.16	0.43 (−0.02,0.41)	0.08
**Peak stiffness**	−0.21 (−0.10,0.01)	0.41	-	-	0.18 (−0.06,0.13)	0.49
**Peak Swelling**	0.01 (−0.07,0.07)	0.98	0.50 (0.39,9.89)	0.04	0.18 (−0.64,1.32)	0.48
**Peak CK**	−0.05 (−0.01,0.01)	0.86	0.33 (0.08,0.44)	0.39	0.28 (−0.03,0.08)	0.28

RF, rectus femoris; PPT, pressure pain threshold; VM, vastus medialis; VL, vastus lateralis; CK, creatine kinase.

## Data Availability

The datasets used and analyzed during this study are available from the corresponding author on reasonable request.
